# Tests of Sight for Signallers and Lookout Men

**Published:** 1882-05

**Authors:** 


RESOLUTIONS ADOPTED BY THE INTERNATIONAL MEDICAL CONGRESS, LONDON, 1881, AS TO "TESTS OF SIGHT SUITABLE TO BE ENFORCED IN THE CASE OF SIGNALLERS AND LOOK-OUT MEN, AND OTHER PERSONS BY LAND OR SEA, WITH SUGGESTIONS AS TO IETERNATIONAL ARRANGEMENTS FOR A UNIFORM SYSTEM OF MARITIME, COAST, AND HARBOR SIGNALLING, WITH A VIEW TO THE SAFETY OF LIFE AND PROPERTY, Followed by Explanatory Remarks under the several Articles.

A.—WITH RESPECT TO LAND.

(1) That the recommendations of the last International Medical Congress, held at Amsterdam in 1879, are accepted by the present Congress as forming the most suitable basis upon which every government may frame its own regulations as to its railway service. They are contained chiefly in Article XII of the "*Projet d'un règlement pour l'examen des facultés visuelles du personnel des chemins de fer*," laid before and accepted by the

Ophthalmological Section of the Amsterdam Congress, which is nearly as follows:

"For admission as *driver* or *stoker*, is required a healthy condition as regards habitual congestion or irritation of the eyes and eyelids; *for each eye*, complete field of vision; normal acuity and refraction; color sense at least four-fifths of the normal; total absence of commencing cataract, or any other progressive disease.

"For admission to other *railway service*, is required a healthy condition as regards habitual congestion, or irritation of the eyes and eyelids; *for each eye*, complete field of vision, total absence of cataract, or any other progressive disease; *for one of the eyes*, normal acuity and refraction, color sense at least three-fifths of the normal; *for the other eye*, sight of at least half the normal, as regards both acuity and color sense."

B.—WITH RESPECT TO SEA.

(2) That in *ocean-going ships* and in *all steamers*, especially those carrying passengers, there should always be in actual control of the helm a person possessing *with the two eyes together, without glasses*, normal sight, both as to acuity and colors; and that, *in addition*, in such ships, *at least one* of the persons actually on the look-out should be similarly qualified.

(3) That, in vessels engaged in the coasting trade, every person liable to take charge of the helm should possess *with the two eyes together*, *without glasses*, sight equal to at least two-thirds of the normal, both as to acuity and colors.

(4) That all persons engaged in marine signalling, ashore or afloat, and all pilots, should have normal sight, both as to acuity and colors, as defined in Article 2.

(5) That hypermetropic persons, although satisfying the requirements of Articles 2, 3, and 4, should, nevertheless, not be admitted, if before the age of eighteen they have a manifest hypermetropia of one dioptre.

(6) That re-examinations should be made at the age of forty-five.

(7) That the examinations should be conducted by persons of recognized competency, under the direction of a central medical authority in each country.

(8) That an international commission should be constituted, to fix upon such further measures as to signals as may be necessary for safe navigation, and, specially, upon the standard colors, and the sizes of the signals employed.

### EXPLANATORY REMARKS.

[THE NUMBERS REFER TO THE RESOLUTIONS.]

### A.—AS TO LAND.

(1) The signal services on land, though quite as important, are not so purely an international matter as those having reference to the sea. Many countries have already established legislation in this respect, and others are introducing it by degrees. It is believed that the standards now recommended may be of general utility, especially in the case of conterminous countries. The mode of examination, for colors especially, is here, as at sea, of the highest possible importance.

### B.—AS TO SEA.

It is obvious that regulations having an international character become every year more urgently required, from the increasing number, size, and speed of vessels.

In view of the practical difficulties with which all compulsory examinations are attended, it has been sought,—

(*a*) To limit the examination in each case to what is strictly necessary.

(*b*) To require them only when absolutely indispensable, and of the smallest possible number of persons.

(*c*) To simplify the methods as much as possible.

On large ships many sailors, not required for the helm, or to be responsible for the look-out, may be admitted without certificate of examination; but as it will be in the interest of all to be possessed of such a certificate, which would represent a higher competency, it may be expected that many would themselves seek for it, from whom it would not necessarily be demanded; and facilities for obtainining it should at all times be at hand in maritime ports.

(2) Good sight *without the aid of* glasses is required, because glasses fail to help just where clear sight is most needed, *e.g.*, in storm, rain, or fog.

*Acuity of Sight.*—Complete acuity is not more than sufficient, and even scarcely sufficient, having regard to the increasing number, size, and speed of steamers. But it will be practically enough if at sea this complete acuity is attained by the use of *both eyes combined.* The number of persons excluded under this rule will be much less than if complete acuity for *each eye separately* is exacted.

The acuity is supposed to be determined by viewing letters or signs at a certain distance, under a certain angle, on the principle of the test-types of Snellen.

*Color Sense* is supposed to be tested by pseudo-iso-chromatic tables, on the principle of those of Stilling, subject to control by the use of light transmitted through colored glass, in imitation of signal lights. This control will also aid in detecting central scotoma for colors, in the very rare cases where it might co-exist with the required acuity.

Holmgren's excellent tests have been already extensively adopted. But their use demands more skill in the examiner. Tests well selected on the principle of Stilling might be very well adopted as standards for ascertaining normal color sense, as well as definite degrees of color sense below the normal. The principle of Stilling has been recommended as affording a quantitative as well as a rapid qualitative test.

(3) A lower standard is fixed in the coasting trade (excluding steamers), because the vessels are smaller and the speed less. Moreover, a demand for full acuity would render it difficult to procure a sufficient number of sailors; as each hand must be liable in small vessels to serve at the helm.

(4) It is obvious that the persons here named must have full acuity and color sense.

(5) Persons having a manifest hypermetropia above that here indicated would not possess, at the age of thirty-five or forty, without glasses, the needful degree of acuity; it is better, then, both for themselves and the service, that they should not be admitted at all.

(6) The attendant practical difficulties have caused one re-examination only to be advised, at the age of forty-five. It has been found that the very great majority of persons, once admitted as having good sight, have retained it up to that age. A great number, no doubt, have been admitted hitherto without sufficient examination. Still, it would not be practicable to institute a general examination of those already in the service. Nevertheless, it would be desirable to examine anew, in the case of passenger steamers, all those responsible as helmsmen and look-out men.

The Congress recommends that surgeons of ships should be qualified to exercise special surveillance as to the sight of those employed in these capacities on board.

(7) A central medical authority is requisite to insure the perfection of the system and its uniformity. He should propose the examiners, and be responsible for their fitness. They should be men of ascertained competency, and as far as practicable, qualified as medical specialists.

(8) The measures recommended in Articles 2 to 7 should be brought into operation without delay. But an international commission would still have to determine the precise color of the glass, securing uniformity in that as well as in the size and disposition of the signal lights.

The Congress lay the greatest stress upon the appointment of this commission in respect of marine signalling, as quite indispensable for the attainment of the object in view. The commission would have to inquire into, and decide upon, many matters on which information is at present incomplete, and regarding which only a few points have been touched upon in Article 8.

Every government, especially the maritime governments, should be requested to place one or more members on the commission, and chiefly experienced naval officers and medical specialists.

It is understood that this question of an international commission is about to be submitted to the legislature of the United States of America, supported by a petition largely signed by scientific men of that country.

The resolutions emanated from the Ophthalmological Section of the Congress, and were drawn up, in the first place, by a committee representing twelve different countries.

FORTY-SEVENTH CONGRESS, FIRST SESSION. HOUSE OF REPRESENTATIVES. REPORT NO. 445.

*The Commitee on Naval Affairs, to whom was referred joint resolution No.* 24, *relating to color-blindness and visual acuteness in persons employed in the Navy and merchant marine, have considered the subject, and submit the following report:—*

At the last session of Congress this subject was brought to the attention of the House by petitions signed by numerous highly distinguished scientific men of the country. The Committee on Naval Affairs of that House gave the subject careful attention, and made a report, which we quote as the best presentment of our views which we are able to make. That report is No. 1569, Forty-sixth Congress, second session.

Dr. B. Joy Jeffries, of Boston, who has devoted much time to the study of this very important subject, and who has voluntarily, and at his own expense, done much to awaken interest in it in several of the States, and who by his disinterested efforts has induced many of the great railroad companies to adopt his system of examination and test for their employés, was invited to come before the committee and give his views upon the subject. He accepted the invitation, and on the twenty-third day of January last he appeared before the committee and illustrated the various methods which have been adopted in this country and in Europe for the detection of color-blindness, and the tests made use of to determine visual power and acuteness, and explained the importance of establishing by an international congress a universal system of examination and tests for these visual defects, and a standard of colors for signals at sea, to be used by maritime nations. An abstract of the remarks of Dr. Jeffries is hereto annexed as an appendix to this report.

The committee think the subject is of so great importance as to demand the attention of all maritime countries, and they believe that the action recommended by this committee will, if adopted by Congress, meet with ready response from the other nations, and that a system may result from it which will prove of vast benefit to the human race.

They recommend the adoption of the accompanying joint resolution as a substitute for that referred to the committee :

FORTY-SEVENTH CONGRESS, FIRST SESSION. HOUSE RESOLUTION 135.

Joint resolution relating to color-blindness and visual acuteness in persons employed in the Navy and merchant marine.

WHEREAS, it is important that definite and uniform standards of examination for color-blindness and tests for visual acuteness in persons employed in the Navy and merchant marine should be established, which shall be in harmony with such standards and tests as are or may be established by other nations. Therefore,

*Resolved by the Senate and House of Representatives of the United States of America, in Congress assembled*, That the President is hereby authorized and directed to appoint, by and with the advice and consent of the Senate, some suitable person qualified for such service, who, with one line officer of the Navy and one medical officer of the Navy, to be detailed or designated by the Secretary of the Navy, shall attend and represent the United States in any international congress or convention held by authority of law in any European nation to consider and act on said subject; and the sum of ten thousand dollars is hereby appropriated, out of any money in the Treasury not otherwise appropriated, to be used and expended, or so much thereof as may be needed, under the direction of the President, to compensate the person so appointed, and defray the necessary expenses of the person so appointed and of the officers of the Navy so detailed or designated. And the person so appointed and the officers of the Navy so detailed or designated under the provisions of this act, shall join in a report of the proceedings of such congress or convention, and the conclusion reached thereby, if any, to the President, to be by him laid before Congress, to the end that an international system of examinations for color-blindness and tests for visual acuteness, and standards for colors for signals used at sea, may be established by law.

